# Lack of Information on the Effects of COVID-19 on Rare Pathologies Has Further Hampered Access to Healthcare Services

**DOI:** 10.3389/fpubh.2022.852880

**Published:** 2022-03-30

**Authors:** Emilia Severin, Dorica Dan

**Affiliations:** ^1^Genetics Department, Carol Davila University of Medicine and Pharmacy, Bucharest, Romania; ^2^Romanian Prader Willi Association, Romanian National Alliance for Rare Diseases RONARD, Zalau, Romania

**Keywords:** rare disease, COVID-19 pandemic, lockdown, digital technology, telemedicine

## Introduction

First, an overview of the Romanian country profile to better understand the context in which our community of rare diseases has developed. Romanian population is just over 19 million people, more than 50% living in the urban area. Romania is one of the new 13 countries that joined the EU. The EU policy in the rare diseases (RDs) field impacted significantly on the Romanian health care policy landscape. So, new strategic directions and priorities for health system reform had to be established, including RDs (1). At the beginning of the pandemic, in March 2020, a health policy for RDs, a national plan for rare diseases and national healthcare programs for rare diseases (dietary and curative treatments), 24 centers of expertise dedicated to certain rare pathologies, the national network of medical genetics, and case management programs integrated into the community health nursing were active. The main organizer of the activities in the field of rare diseases was Romanian Alliance for Rare Diseases (RONARD). The total number of Romanians living with a rare disease is estimated to be up to 1 million, both children and adults. This estimate shows that while individual diseases may be rare, collectively are common. Thus, “the paradox of rarity” highlights rare conditions as a priority of the public health care system in Romania.

## Romania Rare Disease Profile

If we are looking for numbers on a specific RDs, we can check the number of people enrolled in national programs. In Romania, the top three orphan diseases are hemophilia (around 1,500 cases) followed by amyotrophic lateral sclerosis (660 cases) and cystic fibrosis (around 525 cases, 400 children, and 125 adults) as reported by the National Health Insurance House (2). In terms of the age distribution, rare disorders were most frequently represented in the age group of 1–14 years. One of the new National Plan for RDs (spanning the years 2021–2027) priorities is to establish the national registry for all RDs. It will offer epidemiological data required to support the allocation of an adequate budget for a better healthcare organization/planning and sustainable health and social services. Furthermore, the national registry serves as a recruitment tool for the launch of studies focusing on disease etiology, pathogenesis, diagnosis, or therapy. People with a rare disease and their families often have difficulty accessing all aspects of daily life, facing not only the suffering caused by the disease but also the late diagnosis, treatment inadequate, inefficient health care, lack of medical information about the disease, and lack of experts, insufficient social support, social isolation, and financial problems.

## Promoting Action Plan For Rare Disease Community in Times of Pandemic

The sudden arrival of the global COVID-19 pandemic has negatively affected Romanian people with a rare disease and their quality of life related to physical health, emotional health, availability of supplies, access to care, or other specific problems. Hospitals, clinics, or centers of expertise for rare diseases had to close or to reduce their healthcare services. The healthcare system focused on the battle with Covid-19 pathology. For people living with a rare disease, as non-Covid-19 patients, their safe medical care and all aspects of daily life have been disrupted (3).

A survey conducted by RONARD (4) identified significant negative impacts, such as disruption of health care continuity, while appointments, diagnostic protocols, medical treatments, and rehabilitation services were canceled, reduced, or postponed. The results of the survey show that 42% of patients completely (22%) or partially (20%) gave up treatment administered in a hospital, and 2% received medical treatment or rehabilitations therapies in another hospital (COVID-FREE) than usual. Likewise, treatment changes were switched from hospital to home therapy, and difficulties in getting the necessary medicines (prescription only by e-mail) were noticed. Furthermore, Covid 19 risk kept the patients away from seeking treatment in hospitals or ambulatories. EURORDIS (2020) reported similar care disruptions in many European countries based on the results of a multi-country survey (5).

Patients and their caregivers or families had to face a lack of relevant information on how COVID-19 will affect them individually, how to prevent or control exposure to the virus. So, stress, concerns, anxiety, and social isolation heightened. Rare disease people tried to get information using digital technologies and telecommunications, but it was difficult to find specific information on how to be prepared and seek proper care under these unusual circumstances, especially during the first wave of the pandemic (6). Besides, some people from rural areas experienced limited financial resources, digital technology, and internet access. On the other hand, the Coronavirus total lockdown reshaped the family relationships forcing people to live together within a limited space. The results of the RONARD survey showed a positive impact on family relationships.

## Discussion

Hedley et al. (7) consider that the impact of COVID-19 on nations worldwide is likely to push rare diseases further down the queue in terms of national priorities. This would be very damaging, potentially, as the COVID-19 has in fact already served to further illustrate the vulnerability of the rare disease population (7). This statement is true. The COVID-19 pandemic highlighted the vulnerability of the healthcare system and raised new challenges for RDs patients who need to manage their complex conditions requiring coordination across multiple specialties.

The pandemic “opened the doors” for more remote healthcare finding virtual and digital solutions to delivery consultations and e-prescriptions, case management or certain follow-up rehabilitation/therapy sessions. In response to the Covid-19 crisis, in November 2020, Romanian healthcare authorities put in place the national framework legislation to implement Telehealth and Telemedicine solutions for rare diseases community. Telehealth services include various clinical and non-clinical activities, such as medical care, provider and patient education, health information services, and self-care *via* telecommunications and digital communication technologies. Telemedicine provides remote clinical services to patients, such as teleconsultation, tele-expertise, tele-assistance, teleradiology, telepathology, and telemonitoring. Telemedicine solutions offer both benefits (real-time encounters, comfort, lower costs, and control the exposure to pathogens) and limitations for RDs people. Despite its benefits, not all remote healthcare services fit for all purposes (8), and not everyone can use technology.

The experience gained in the COVID-19 pandemic, both by patients and by the Centers of Expertise for RDs, draws attention to the need to update and harmonize the legal framework in the fields of health, social care, and education. Learning from the INNOVCare Project Recommendations, Castro et al. (8) conclude that national health and welfare systems need to become more coordinated and resilient, always ensuring integrated care and even more during a crisis (8).

## Author Contributions

ES and DD designed the survey, discussed the results, and contributed to the final manuscript. All authors contributed to the article and approved the submitted version.

## Conflict of Interest

The authors declare that the research was conducted in the absence of any commercial or financial relationships that could be construed as a potential conflict of interest.

## Publisher's Note

All claims expressed in this article are solely those of the authors and do not necessarily represent those of their affiliated organizations, or those of the publisher, the editors and the reviewers. Any product that may be evaluated in this article, or claim that may be made by its manufacturer, is not guaranteed or endorsed by the publisher.
